# Rationale for Combining Radiotherapy and Immune Checkpoint Inhibition for Patients With Hypoxic Tumors

**DOI:** 10.3389/fimmu.2019.00407

**Published:** 2019-03-12

**Authors:** Franziska Eckert, Kerstin Zwirner, Simon Boeke, Daniela Thorwarth, Daniel Zips, Stephan M. Huber

**Affiliations:** ^1^Department of Radiation Oncology, University Hospital Tuebingen, Tuebingen, Germany; ^2^German Cancer Consortium (DKTK) Partnersite Tuebingen, German Cancer Research Center (DKFZ), Heidelberg, Germany; ^3^Section for Biomedical Physics, Department of Radiation Oncology, University Hospital Tuebingen, Tuebingen, Germany

**Keywords:** immunotherapy, radiotherapy, hypoxia, T cells, cancer, T_reg_s, immune checkpoint inhibition

## Abstract

In order to compensate for the increased oxygen consumption in growing tumors, tumors need angiogenesis and vasculogenesis to increase the supply. Insufficiency in this process or in the microcirculation leads to hypoxic tumor areas with a significantly reduced pO2, which in turn leads to alterations in the biology of cancer cells as well as in the tumor microenvironment. Cancer cells develop more aggressive phenotypes, stem cell features and are more prone to metastasis formation and migration. In addition, intratumoral hypoxia confers therapy resistance, specifically radioresistance. Reactive oxygen species are crucial in fixing DNA breaks after ionizing radiation. Thus, hypoxic tumor cells show a two- to threefold increase in radioresistance. The microenvironment is enriched with chemokines (e.g., SDF-1) and growth factors (e.g., TGFβ) additionally reducing radiosensitivity. During recent years hypoxia has also been identified as a major factor for immune suppression in the tumor microenvironment. Hypoxic tumors show increased numbers of myeloid derived suppressor cells (MDSCs) as well as regulatory T cells (T_reg_s) and decreased infiltration and activation of cytotoxic T cells. The combination of radiotherapy with immune checkpoint inhibition is on the rise in the treatment of metastatic cancer patients, but is also tested in multiple curative treatment settings. There is a strong rationale for synergistic effects, such as increased T cell infiltration in irradiated tumors and mitigation of radiation-induced immunosuppressive mechanisms such as PD-L1 upregulation by immune checkpoint inhibition. Given the worse prognosis of patients with hypoxic tumors due to local therapy resistance but also increased rate of distant metastases and the strong immune suppression induced by hypoxia, we hypothesize that the subgroup of patients with hypoxic tumors might be of special interest for combining immune checkpoint inhibition with radiotherapy.

## Introduction

Solid tumors are prone to encounter chronic or intermittent hypoxic microenvironment. Hypoxia results from an imbalance of O_2_ consumption by the tumor and O_2_ delivery by perfused tumor vessels. The latter is limited since tumor vasculogenesis and angiogenesis usually lags behind expansion of tumor mass. In addition, tumor vessels often show aberrant architecture, may have dilated or blind-ending lumina, and lack normal vessel walls (1). As a consequence, increasing intra-tumoral pressure may compress the vessel lumen accentuating malperfusion of the tumor. Concomitant to insufficient O_2_ and nutrient supply, this malperfusion restricts delivery of systemically administered drugs such as chemotherapeutics or immunomodulating antibodies limiting the efficacy of these therapies in hypoxic tumor areas (2). Beyond that, hypoxia attenuates DNA damages conferred by ionizing radiation.

Oxygen tensions vary considerable in areas of diffusion-limited chronic hypoxia or perfusion-limited cycles of intermittent hypoxia and reperfusion, hence, triggering a plethora of different cellular adaptation processes (3). Oxygen-sensing processes comprise stabilization of hypoxia-inducible factor (HIF), nutrient depletion-induced down-regulation of the mTOR (mammalian target of rapamycin) pathway (4), impairment of oxidative folding of proteins in the endoplasmic reticulum and unfolded protein response (5), DNA replication stress (6), or oxygen-dependent remodeling of chromatin (7–9). Adaptations to hypoxia include metabolic reprogramming that maintains structural integrity (10), as well as energy (4), redox (11, 12), pH (13), and lipid (14) homeostasis of the hypoxic tumor cell. These complex adaptations, however, induce tumor heterogeneity and may be accompanied by adoption of more malignant phenotypes (15).

Therefore, intratumoral hypoxia has major implications in cancer biology and treatment resistance. Based on the knowledge of an increased radioresistance of hypoxic cancer cells and impaired prognosis for patients with hypoxic tumors, imaging modalities for hypoxia and treatment strategies to overcome the disadvantages of hypoxia have been developed in radiation oncology. With the rise of immunotherapy in cancer over the recent years and the establishment of immune checkpoint inhibition as a standard treatment for several cancer entities, well-known concepts in cancer and radiobiology have been evaluated for their effects on immune responses to cancer. For hypoxia, pronounced immunosuppressive properties have been described by several groups. This article aims at giving an overview and converging the knowledge about tumor hypoxia in the context of radiotherapy and immunotherapy of cancer patients, hypothesizing that patients with hypoxic cancers might benefit most from combination treatments in curative treatment settings.

## Hypoxia-Associated Malignant Progression of Tumor Cells

Master regulators of metabolic reprogramming under hypoxia are the O_2_-sensitive hypoxia-inducible transcription factors (HIFs), the cellular nutrient sensing mTOR and the energy-sensing AMP kinase, as well as the unfolded protein response. They induce downregulation of anabolic metabolism, up-regulation of nutrient import and glycolysis, a switch from oxidative phosphorylation to lactic acid fermentation, up-regulation of acid extrusion pathways such as monocarboxylate transport, adaptation of glutamine metabolisms to maintain fuelling of the citrate pool, alteration of lipid metabolism, attenuation of mitochondrial reactive oxygen species (ROS) formation and/or up-regulation of oxidative defense [for recent reviews (4, 16, 17)].

**Figure 1:**
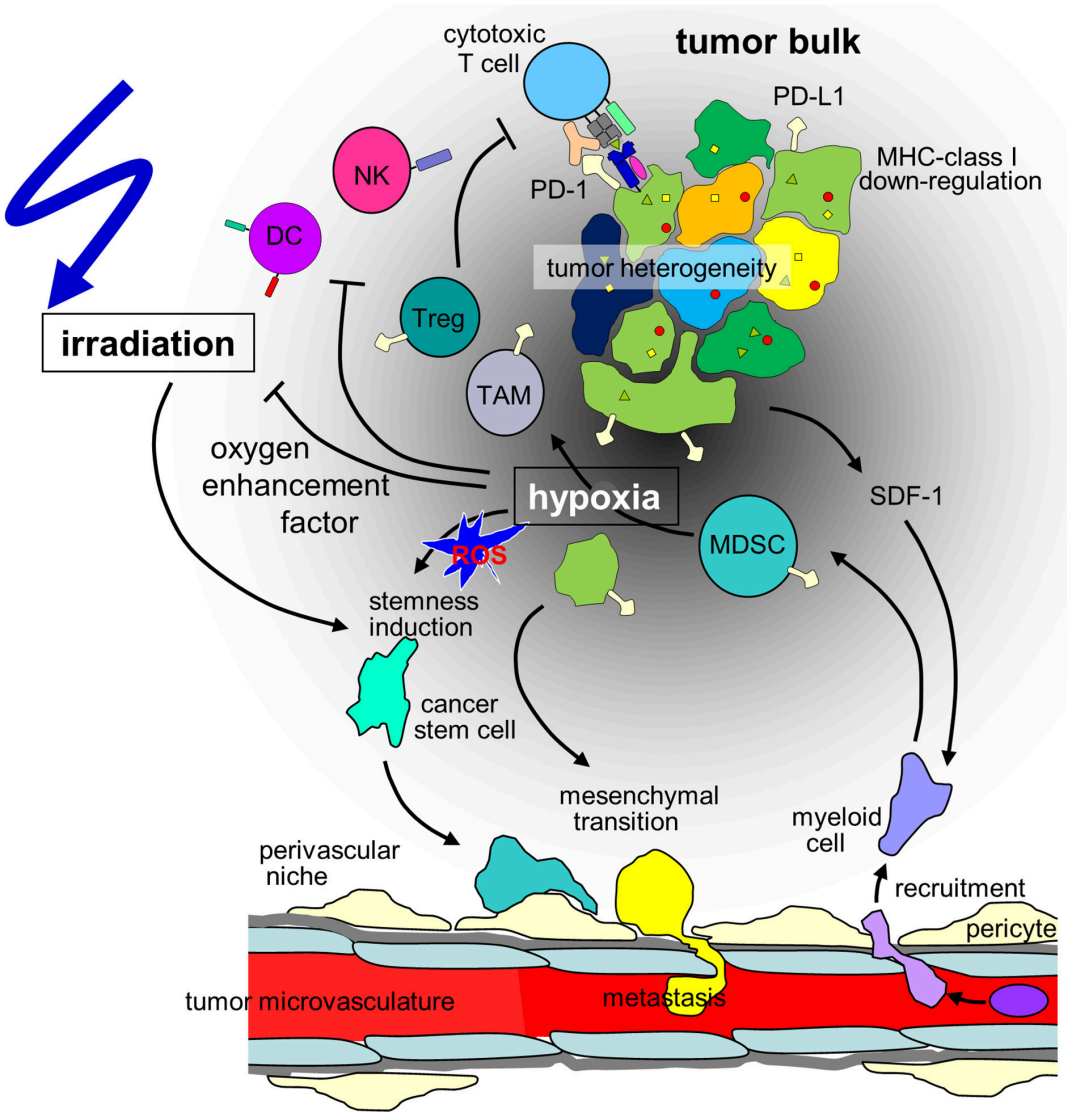
Hypothesis of the influence of hypoxia on cancer cells and the immune microenvironment in the context of radiotherapy of solid tumors. Hypoxia may stimulate in a subset of tumor cells mesenchymal transition and metastasis or induction of cancer stem(-like) cells. The radioresistant phenotype of the latter together with the decline in radiation-induced DNA damage with decrease in oxygen tension (oxygen enhancement factor) contribute to the radioresistance of hypoxic tumors. Moreover, hypoxia/radiation-induced migration may lower locoregional tumor control by radiotherapy. In addition, tumor hypoxia recruits immunosuppressive cell types such as regulatory T cells (T_reg_s) and myeloid derived suppressor cells (MDSCs) that mature to M2-polarized tumor associated macrophages (TAMs) via stromal cell-derived factor-1 (SDF-1) chemokine signaling. Dendritic cell (DC) function is modulated to T_H_2 polarized immune responses which suppress anti-tumor immunity. Finally, hypoxia may induce downregulation of MHC class-I molecules and Natural Killer (NK) cell-activating ligands and upregulation of programmed death-ligand-1 (PD-L1) on tumor cells. (ROS: reactive oxygen species).

Metabolic reprogramming may be paralleled by a HIF-regulated phenotypic switch leading to cellular plasticity of tumor and stroma cells which drives tumor heterogeneity. In particular, a hypoxic microenvironment may stimulate in a subset of tumor cells neuroendocrine differentiation, epithelial-mesenchymal transition (EMT) (or neural/glial-mesenchymal transition in brain tumors) or induction of cancer stem (-like)/tumor initiating cells (CSCs) (11). Signaling cascades that induce CSC phenotypes in distinct hypoxic niches are probably triggered by ROS that are formed during the metabolic adaptation to hypoxia (Figure 1). Notably, EMT and CSC induction seems to be highly interrelated and involve HIF signaling [for review see (18, 19)]. Importantly, EMT and upregulation of CSC properties are accompanied by a change from a “grow” to a “go” phenotype. As a consequence, hypoxic tumors are at higher risk of tissue infiltration and metastasis (18, 19).

Moreover, hypoxia and in particular ROS formation during reoxygenation have been shown to favor genetic instability and to increase mutagenesis in tumors by induction of DNA damage and/or deregulation of DNA damage response and apoptotic pathways fostering malignant progression of tumor cells (10, 11). Notably, genetic instability has been associated with response to immune checkpoint inhibition on the one hand and decreased tumor immunogenicity by formation of immune-evasive subclones on the other hand (20, 21). Beyond malignant progression and immune evasion, hypoxia confers resistance to chemo- (2) and radiation therapy as described in the next paragraphs.

## Radioresistance of Hypoxic Tumor Cells

About half of all cancer patients undergo radiation therapy often applied in fractionated regimens. Conceptually, a radiation dose of 1 Gy with high energy photons causes about 20 DNA double strand breaks (DSBs) per nucleus on average in normoxic tissue (22). Nuclear DNA DSBs have been proposed to be most hazardous for the cell since when left unrepaired they inevitably provoke chromosome aberrations in mitosis. Tumors are thought to become eradicated if the quantity of radiation induced DSBs exceeds the capacity of DNA DSB repair by non-homologous end joining in G1 phase of cell cycles and additional homologous recombination in S and G2 phase (23). Hypoxia has turned out to be a negative predictive factor for the response to radiation therapy (24) due to lowering the efficacy of ionizing radiation by a factor of 2–3. Mechanistically, this so-called oxygen enhancement ratio (OER) most probably reflects three processes in irradiated cells: O_2_ fixation of DNA damages, O_2_-dependent formation of ROS by the mitochondria, as well as hypoxia-induced acquisition of a radioresistant phenotype.

### O_2_ Fixation of DNA Damages

Radiation therapy damages cells by ionization of molecules. Among those, H_2_O with the far highest concentration (more than 50 M) of all molecules in a cell absorbs the largest fraction of the radiation energy. Energy transfer to H_2_O leads to formation of hydrogen (^•^H) and hydroxyl radicals (^•^OH) in a process referred to as radiolysis of H_2_O. Formation of ^•^H radicals has been proposed to confer reductive stress to the irradiated cells (25) while the high reactivity and low lifetime of ^•^OH radicals may remove hydrogen atoms from neighboring macromolecules resulting in formation of macromolecule radicals. With a lower stochastic probability formation of macromolecule radicals also occurs upon direct absorption of radiation energy by the macromolecules. Now, the O_2_ tension comes into the play. Under normoxia, at high O_2_ partial pressure in the cell, the radical atom within the macromolecule has been suggested to become oxidized which may be associated with the cleavage of molecular bonds of the macromolecule. Under hypoxia, however, at low cellular O_2_ tension and reductive cellular redox state (which comprises a high ratio between reduced and oxidized glutathione and a high capacity of oxidative defense), macromolecule radicals have been proposed to become “repaired” chemically (Figure 1).

Thus, a high O_2_ tension may evoke DNA strand breaks whenever radiation-induced radical formation occurs within the phosphate deoxyribose backbone of the DNA. If radical formation concurs in close vicinity in both anti-parallel DNA strands, high oxygen pressure promotes formation of DNA DSBs. This so-called oxygen fixation hypothesis which was developed in the late 1950's, however, explains only insufficiently the oxygen enhancement ratio in radiation therapy. It neither considers hypoxia-mediated effects on DNA repair (26) nor radiation-induced secondary cell damages by mitochondrial ROS formation. The latter are also highly O_2_-dependent as discussed in the following paragraphs.

### Mitochondrial ROS Formation

Early microbeam technologies which allow irradiation of cellular substructures provided strong evidence for a much higher efficacy of ionizing radiation when the nucleus was targeted as compared to selective irradiation of the cytoplasm (27). Therefore, as central dogma of radiation therapy, the genotoxic effects of radiation has been attributed for many years to an interaction between ionizing radiation and the nucleus as primary mechanism (25). Notwithstanding, more recent work, however, suggests that nuclear DNA damage does not exclusively require irradiation of the nucleus and even can be observed in unirradiated bystander cells [for review see (28)]. Notably, inhibiting ROS formation reportedly prevents nuclear DNA damage of the beam-targeted and the bystander cells (29) indicating ROS mediated spreading of the absorbed radiation energy. Furthermore, experiments comparing cells with mitochondrial DNA-proficient (ρ^+^) and -deficient (ρ^0^) mitochondria strongly suggest the involvement of mitochondrial electron transport chain in genotoxic damage mediated by radiation (29–33). Most importantly, the fraction of mitochondrial ROS formation-dependent DNA damage has been proposed to increase with O_2_ tension (34).

Mechanistically, ionizing radiation reportedly increase intracellular free Ca^2+^ concentration in several tumor entities such as lymphoma (35), leukemia (36, 37), or glioblastoma (38). Intracellular Ca^2+^ buffering experiments demonstrated that Ca^2+^, in turn, stimulates in the presence of O_2_ mitochondrial ROS formation (30) probably in concert with the transient energy crises observed in irradiated cells (39, 40). Both, low ATP/ADP ratios and high Ca^2+^ concentrations disinhibit mitochondrial electron transport chain, leading to hyperpolarization of the inner mitochondrial membrane potential ΔΨ_m_ which is directly linked to superoxide anion (^•^${\text{O}}_{2}^{-}$) formation by slippage of single electrons to O_2_ [for review see (41)]. Ca^2+^-mediated ^•^${\text{O}}_{2}^{-}$ formation by the electron transport chain, in turn, provokes mitochondrial membrane permeability transition and eventually dissipation of ΔΨ_m_ and mitochondrial disintegration (42). Of note, radiation-stimulated permeability transition of few affected mitochondria and consequent local release of mitochondrial Ca^2+^ has been proposed to stimulate Ca^2+^-overflow, ROS formation, and Ca^2+^ re-release of adjacent mitochondria, thereby propagating radiation-induced mitochondrial ROS formation through the mitochondrial network in a spatial-temporal manner (30).

As a matter of fact, inhibitors of mitochondrial permeability transition blocked radiation-induced mitochondrial ROS formation (30) and in some but not all cell lines O_2_-dependent radiosensitivity (43). Combined, these observations strongly suggest that O_2_ tension-dependent mitochondrial ROS formation and adjunct DNA damage contribute significantly to the OER phenomenon. Beyond stimulation of mitochondrial ROS formation, radiation has been reported to up-regulate activity of uncoupling proteins (UCPs) in the inner mitochondrial membrane (34). UCPs shortcircuit ΔΨ_m_ thereby directly counteracting radiation-stimulated mitochondrial ROS formation [for review see (41)]. As described in the next paragraph, adaptation to hypoxia may also involve up-regulation of mitochondrial uncoupling.

### Radioresistant Phenotypes Induced by Hypoxia

Adaptation of cells to hypoxia has been described for highly oxidative phosphorylation-dependent normal proximal tubule cells. By repeatedly subjecting these cells to hypoxia and re-oxygenation cycles over weeks strong up-regulation of oxidative defense and mitochondrial uncoupling was induced. Besides diminishing reoxygenation-induced ΔΨ_m_ hyperpolarization, ^•^${\text{O}}_{2}^{-}$ formation, and consecutive cell damage, mitochondrial uncoupling confers cross-resistance to ionizing radiation (44). Importantly, tumors such as proximal tubule-derived renal clear cell carcinoma show high upregulation of mitochondrial uncoupling proteins (44) pointing to hypoxia-induced mitochondrial uncoupling as one potential mechanism of induced resistance *in vivo*. Similarly, cyclic hypoxia and reoxygenation reportedly upregulates *in vitro* the mitochondrial citrate carrier SLC25A1 in cancer cell lines that contributes to an increased radioresistance-conferring oxidative defense (11). Beyond that, further metabolic pathways up-regulated in hypoxic cells such as glutamine-dependent glutathione formation (12) or glycolysis-associated pyruvate accumulation [for review see (4)] result in increased capacity of radical scavenging that may confer radioresistance.

Moreover, the above mentioned hypoxia-triggered induction/selection of CSCs reportedly associates with an increased intrinsic radioresistance (Figure 1). CSCs have been supposed to express higher oxidative defense, pre-activated and highly efficient DNA repair and anti-apoptotic pathways rendering them less vulnerable to ionizing radiation [for review see (18)]. Beyond that, CSCs may overexpress certain Ca^2+^ and electrosignaling pathways that improve stress response upon irradiation (45, 46) as demonstrated for the mesenchymal subpopulation of glioblastoma stem cells (47).

Finally, at least in theory, the above mentioned hypoxia-induced migratory phenotype of tumor cells might limit efficacy of radiotherapy in fractionated regimens. One might speculate that highly migratory cells evade from the target volume covered by the radiation beam. In glioblastoma, stabilization of HIF-1α stimulates auto/paracrine SDF-1 (CXCL12)/CXCR4-mediated chemotaxis the programming of which strongly depends on electrosignaling as one key regulator of chemotaxis (48). Likewise, ionizing radiation stimulates the same pathways also by activating the HIF-1α/SDF-1/CXCR4 axis (48). It is, therefore, tempting to speculate that hypoxia and radiation cooperate in stimulating hypermigration during fractionated radiotherapy. Evidence, however, that hypermigration indeed has any relevance for local tumor control by radiation therapy in the clinical setting is missing. Nevertheless, tumor hypoxia is a severe obstacle of radiation therapy. The next section deals with concepts of visualization and effective treatment of hypoxic tumors for radiation therapy.

## Treatment Modifications Targeting Hypoxia in Radiation Oncology

Cellular effects on radiation-response under hypoxia *in vitro* (49, 50) cannot be directly transferred to xenografts *in vivo* and tumors in patients. The OER (determined to be 2–3 *in vitro* (51), as described above) seems to be lower *in vivo*. This is on the one hand due to the fact that parts of the tumor volume are sufficiently oxygenated since oxygen tension is decreasing only gradually around perfused blood vessels (52–54). On the other hand, depending on the tumor entity, decrease of the bulk tumor mass during fractionated radiation may lead to tumor reoxygenation (55, 56). Extensive research on the tumor microenvironment (hypoxia, vasculature, necrosis and metabolism) and its impact on radioresistance has been done in *xeno*graft models for head and neck squamous cell carcinoma (HNSCC), glioblastoma, non-small cell lung cancer (NSLCL) and colorectal carcinoma and sarcoma cell lines (51, 57–61). *In vivo* models were also used to show the predictive value of functional tumor imaging with hypoxia sensitive tracers for positron emission tomography (PET) imaging (62–64). Based on hypoxia imaging, different approaches including dose escalation, HIF1α-inhibitors, hypoxia activated prodrugs and hyperbaric oxygen (HBO) or carbogen breathing were studied to overcome treatment resistance with promising results (65–67).

In a clinical setting of HNSCC and cervix cancer, an association between oxygen tension and radioresistance could be shown. For 35 patients with locally advanced HNSCC invasive pO2-measurement with oxygen sensitive electrodes with >15% of pO2 values below 2.5 mm HG, was associated with reduced local control at 2 years (68). In a prognostic validation study as well as in a multicenter study with more than 390 patients, the results could be confirmed (69). There are matching results of worse prognosis for patients with cervical cancer with decreased pO2 values before radiotherapy (70, 71). With advances in imaging methods, non-invasive measurement of hypoxia, based on positron emission tomography (PET) with different hypoxia specific tracers, e.g., [^18^F]fluoromisonidazole (FMISO), [^18^F]fluoroazomycin arabinoside (F-AZA), [^18^F]fluortanidazole (HX4) and [^64^Cu]diacetyl-bis(N^4^-methylthiosemicarbazone (Cu-ATSM), and magnetic resonance imaging (MRI) were established and could be correlated to outcome in HNSCC, cervical cancer and NSCLC (72–81). Hypoxia imaging is also closely related to other functional imaging modalities such as FDG-PET or functional MRI (82–84). Based on this evidence, there were major efforts to target hypoxia in the curative setting of radiotherapy during the last decades.

In parallel to the findings of hypoxia as a common phenomenon in solid tumors in the fifties, efforts were started to increase tumor oxygenation by HBO treatment under 2 to 4 atmospheres (85). Due to small numbers of patients in these trials and difficulties of irradiation in pressure chambers, the promising results could not advance into clinical use. Inhalation of carbogen with nicotinamide was the topic of a large phase III trial, which showed decreased regional failure (86). Another approach is the use of hypoxia specific agents like nitroimidazoles. In a trial of The Danish Head and Neck Cancer group (DAHANCA 5) the addition of nimorazole to standard treatment showed an increase in locoregional control (LRC) as well as disease-free survival (DFS) for patients with increased osteopontin levels (87) or a specific gene expression profile (88), both linked to hypoxia. Since then nimorazole is standard of care in Denmark during radiotherapy of HNSCC. To evaluate this combined approach, a large European Organization for Research and Treatment of Cancer (EORTC) phase III trial was conducted with results pending (NCT01880359). With the possibilities of modern radiotherapy techniques like intensity modulated radiotherapy (IMRT) and image-guided radiotherapy (IGRT), first trials with dose escalation based on [^18^F]fluorodeoxyglucose (FDG) or FMISO are conducted with conflicting results for toxicity and local control data pending (89, 90). A large meta-analysis of all studies with hypoxic modification in HNSCC of 32 trials with more than 4,800 patients included, showed a significant survival benefit of the intervention vs. the control group (91). In a phase II trial an increased radiation dose could not overcome the worse prognosis of hypoxic NSCLC (92). In summary, the big hopes of targeting hypoxia could not be translated directly into the clinic (93).

## Immunosuppression in the Hypoxic Tumor Microenvironment

Hypoxia in the tumor microenvironment influences the interaction between cancers and the immune system on all levels. Cancer cells regulate the interaction surface with immune cells, the cytokine microenvironment is altered, and immune cell function is reshaped.

### Immune-Relevant Changes in Cancer Cells Under Hypoxia

Cancer cells under hypoxic conditions show a downregulation of MHC class-I molecules (94) (Figure 1), which are crucial for the immune recognition and immune mediated lysis of tumor cells (95). Several immune checkpoints are upregulated in hypoxic conditions. HIF-1α mediates the upregulation of HLA-G (96), which has been described as immunosuppressive (97, 98). In pancreatic cancer HLA-G is a negative prognostic marker, and downregulation of ILT-2 (the receptor of HLA-G) in immune cells activates anti-tumor immunity (99). In addition, hypoxia induces upregulation of CTLA-4 and PD-L1 on tumor cells via HIF-1α in several different mouse and human tumor cell lines (Figure 1). Enhanced PD-L1 abundance could be linked to a HIF-1α binding site in the PD-L1 promotor (100). In renal cell carcinoma elevated PD-L1 levels were correlated with HIF1α levels linked to impaired function of the Von-Hippel-Lindau (VHL) protein (101). In patient samples, HIF1α genes and expression also correlated with PD-L1 expression. The functional link of PD-L1 expression and HIF1α was established by knock-down experiments (101, 102). In hepatocellular carcinoma patient samples PD-L1 expression also was linked to hypoxia and showed prognostic value (103).

Hypoxia has also been linked to downregulation of DNA damage response proteins such as RAD51 in prostate cancer (104), and RAD51 and BRCA1 in breast cancer (105), respectively. BRCA1 downregulation has been shown to be epigenetically regulated in different cancer cell lines (106). Impaired DNA-double-strand-break repair under hypoxic condition might lead to a higher mutation rates and more malignant phenotypes (104). On the other hand, more mutations might also lead to more neoantigens possibly supporting tumor-immune responses. Intriguingly, mutational burden is one of the most promising predictive factor for treatment with immune-checkpoint-inhibition (107). In concordance, the antigenic landscape of prostate cancer is modified by the applied oxygen tension (108) *in vitro*.

### Hypoxic Immune Microenvironment

The immune microenvironment of tumors also undergoes profound changes with the development of intratumoral hypoxia. Hypoxia induced downregulation of ADAM-10 (109) and upregulation of CCL28 (110, 111) and IL-10 (112) all lead to immunosuppression via shedding of MHC class I chain-related molecule A (MICA) and hampering cytolytic action of immune cells, T_reg_ recruitment and enhancing suppressor MDSc, respectively. Hampered anti-tumor immunity in hypoxic tumors is mainly mediated by adenosine receptor signaling (113). Adenosine is formed by hydrolysis of tumor cell-derived ATP in the extracellular space (114). Adenosine receptors are a direct target of HIF1α and have been reported to enable stem (like) cell enrichment in breast cancer (115). Clinical data as well as *in vivo* data in an autochthonous mouse model linked adenosine A2A receptor with carcinogenesis and immune resistance of HNSCC (116). Tumor reactive CD8^+^ cells express A2A receptors and show enhanced activity upon downregulation or blockade thereof (117). Oral A2A receptor inhibitors have been developed and tested preclinically (118). *Ex vivo* testing suggests synergistic effects with immune checkpoint blockade (119).

Consequently, several cell subsets required for efficient anti-cancer immune responses have been described to be impaired or inhibited by hypoxia. Mechanisms of the innate immune system, such as NK cell-mediated killing of cancer cells is disturbed due to downregulation of the respective activating ligands on tumor cells (120). Concerning adaptive immunity, several critical steps are hampered under hypoxic conditions. Dendritic cell function is modulated to T_H_2 polarized immune responses, consequently, T cells primed under hypoxia preferably are T_H_2-polarized and thus suppress anti-tumor immunity (121) (Figure 1). At the same time, the development of anti-cancer T_H_1 cells is inhibited (122) and CD8^+^ effector T cells are inhibited in their proliferative activity under hypoxia, possibly via IL-10 (112).

### Regulatory T Cells

In addition, major immunosuppressive cell types in the tumor microenvironment are upregulated under hypoxic conditions, such as regulatory T cells (T_reg_s) and myeloid derived suppressor cells (MDSCs) and tumor associated macrophages (TAMs) (Figure 1). T_reg_s have been described as major players in cancer immunosuppression by inhibiting effector T cells and fostering angiogenesis (123) and have been described to be increased in hypoxic tumors (124). Several mechanisms for this phenomenon have been proposed. In gastric cancer, FoxP3 (as a marker for T_reg_s) is strongly associated with HIF-1α and TGFβ and acts as negative prognostic factor. *In vitro*, TGFβ blockade diminished the T_reg_ induction under hypoxic conditions (125). This has been linked to hypoxia-induced NANOG expression (126). SDF-1/CXCR4 signaling induced by hypoxia also has been linked to T_reg_ recruitment (127). Another major mechanism described for ovarian as well as for liver cancer is the induction of CCL28. In ovarian cancer CCL28 recruits T_reg_s and leads to accelerated tumor growth *in vitro* as well as in orthotopic models of intraperitoneal tumors (110). These findings have been confirmed for hepatocellular carcinoma (111). The interplay of these different factors for T_reg_ accumulation has not been clarified yet.

### Myeloid-Derived Suppressor Cells (MDSCs) and Tumor Associated Macrophages (TAMs)

Hypoxia leads to the recruitment of MDSCs (128) as well as their accumulation (129) in a hepatocellular carcinoma model as well as in gliomas (130). In the tumor microenvironment MDSCs differentiate to macrophages (131). In hypoxia, macrophages are preferably polarized to the immunosuppressive M2 phenotype (132, 133). M2 macrophages support tumor growth directly (134–136) and simultaneously prevent immune destruction (137, 138). Interestingly, myeloid cells have also been described to be involved in the formation of pre-metastatic niches in secondary organs (139, 140).

## Rationale for Combining Radiotherapy and Immunotherapy

### Immune Checkpoint Inhibition for Cancer Therapy

Immune checkpoint inhibition (ICI) gained increasing interest as a new paradigm in cancer treatment as several encouraging clinical trials were published (141–143). However, in some other studies, ICI showed less promising results (144, 145). There is still a considerable number of patients who do not response at all, solely achieve a partial response or relapse in spite of notable initial response, yet. Several other immunotherapy approaches are being developed (146) [such as cytokine based therapy (147–149) or vaccines (150, 151)], however, the clinical development is most advanced for CTLA-4 and PD-1/PD-L1 blockade.

As reviewed in Wolchok et al. (152) CTLA-4 has been identified as a negative regulator of T-cell activation binding to the B7 protein on antigen presenting cells. This interaction prevents the binding of CD28 to B7, a necessary costimulatory signal for T cell activation following the recognition of respective antigens by the T-cell-receptor representing a very early step in the immune cascade (153). CTLA-4 deficient mice show massive lymphoproliferation, multi-organ tissue destruction and early letality (154). Blockade of CTLA-4 has been shown to induce T cell activation (155, 156) and anti-tumor immunity in preclinical models (157). These findings translated into clinical benefits and long-term cancer control first in patients with malignant melanoma (158, 159). A recent compilation of finished and ongoing clinical trial shows the application of CTLA-4 blockade in numerous cancer entities, therapeutic settings and combinatorial approaches (160).

In clinical cancer therapy, blockade of the PD-1/PD-L1 axis has become even more prominent as indicated by the numbers of ongoing clinical trials (160). The inhibitory effect of PD-1/PD-L1 interaction is predominant during the inflammatory phase in peripheral tissues (161). Similar to CTLA-4, mice deficient for PD-1 developed severe autoimmune symptoms indicating an inhibitory function of PD-1 on immune activation (162). It was soon linked to immune-evasion of tumors as cancer cells show a high expression of PD-L1 and thus directly inhibit T-cell activation in the tumor microenvironment (163). PD-1 also plays a major role in T-cell exhaustion in chronic inflammatory processes and cancer (164). After initial signs of safety and activity of blocking PD-1 for cancer treatment (165), numerous randomized trials have shown clinical benefit of single-agent or combined treatment using PD-1 or PD-L1 antibodies (166).

### Immune Effects of Radiation

Rare abscopal effects (response of distant, non-irradiated lesions) in irradiated patients have been described many years ago [reviewed in (167)], but the interaction of radiation and tumor specific immune responses was increasingly understood later on (168).

**Figure 2:**
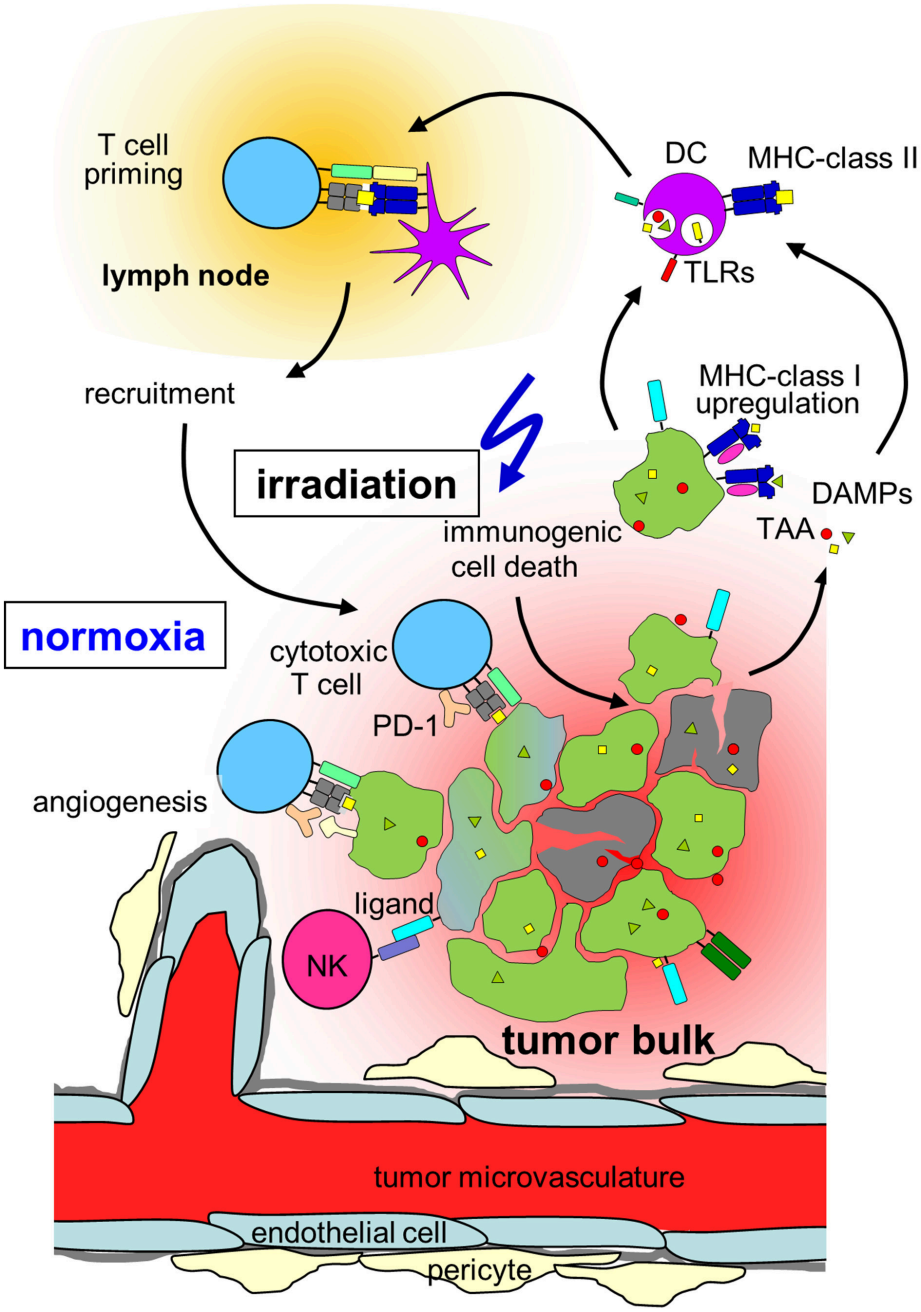
Hypothesis on radiation-induced immunogenic cell death in normoxic tumors. In a normoxic tumor microenvironment, irradiation may lead to effective anti-tumor immune responses by induction of upregulation of MHC class-I on the tumor, immunogenic cell death, release of danger associated molecular patterns (DAMPs) activating toll-like receptors (TLRs) and induction of new tumor associated antigens (TAAs). Maturation of dendritic cells (DCs) and upregulation of MHC-class II is followed by T cell priming in the draining lymph node, cytotoxic T cells and natural killer (NK) cells travel back to the tumor and lead to lysis of tumor cells. Please note, that radiation also induces immunosuppressive processes in normoxic tumors (which are not depicted) such as up-regulation of programmed death-ligand-1 (PD-L1) or T_reg_s (for details, see chapter Immune effects of radiation).

In addition to direct cytotoxic effects of radiotherapy and reoxygenation in solid tumors during fractionated radiation, local irradiation also affects the tumor immune microenvironment. In contrast to the predominant perception of radiotherapy being basically immunosuppressive, several mechanisms have been identified how irradiation might lead to better anti-tumor immune responses as summarized by Demaria and Formenti (169). Radiation influences every step of the “cancer immunity cycle” (170). The cancer cell death induced by irradiation does not only lead to antigen release, but has been characterized as immunogenic cell death characterized by the release of danger signals (171, 172) such as membranous calreticulin exposure and release of HMGB1 and ATP into the extracellular space leading to activation of the innate immune system (173, 174) (Figure 2). Radiation induces upregulation of MHC-I complexes on cancer cells (175) and priming and maturation of antigen-presenting cells (176, 177). After traveling to draining lymph nodes, these antigen-presenting cells are able to prime T cells specific for tumor associated antigens (178). The primed and activated effector T cells show increased infiltration into irradiated tumors (179–181). In addition to the effects on T cell based anti-tumor immune responses, irradiation is able to repolarize macrophages to a tumor inhibiting M1-subtype (182) and activate natural killer cells (183) (Figure 2).

On the other hand (and explaining the scarce clinical evidence for anti-tumor immune induction by radiotherapy alone) irradiation induces immunosuppressive mechanisms in solid tumors (184). One major mechanism is the upregulation of PD-L1 in irradiated tumors (185–187). Even combined treatment of CTLA-4 blockade with irradiation led to upregulated PD-L1 level and treatment resistance, which could be overcome by adding PD-1/PD-L1 blockade to the regimen in a preclinical model (188). In addition, radiation leads to the accumulation of T_reg_s (189, 190) as well as the release of immunosuppressive molecules such as TGFβ (191, 192). Curative, normofractionated radiotherapy leads to significant changes in the peripheral immune status of the patients with a decrease of naïve CD4^+^ lymphocytes and an increase in T_reg_s (193–195). These findings led to the rationale of combining cancer radiotherapy with immune checkpoint inhibition (196).

### Combined Radiation and Immune Checkpoint Inhibition

The rationale of combining immunotherapy and radiotherapy has been discussed intensely in several review articles [e.g., (197, 198)]. Initial clinical signs of synergistic and abscopal effects after combination therapy of radiotherapy and immune checkpoint inhibition were reported in a patient with malignant melanoma who had progressed on Ipilimumab but showed a second systemic response after palliative radiotherapy for a paraspinal lesion (199). Initial phase II studies in melanoma showed an abscopal response rate of 18% (200). Immune checkpoint inhibition has been combined with palliative radiotherapy (201) as well as with ablative stereotactic irradiation (202). Furthermore, a recent trial in stage III non-small cell lung cancer encourages efforts of combining both therapeutic strategies in curative settings as well (203). Here, Durvalumab (a monoclonal PD-L1-antibody) consolidation after definitive radiochemotherapy showed significantly prolonged progression-free survival rates and increased overall survival compared to the placebo group with short time between end of radiochemotherapy and start of checkpoint-blockade showing an even larger effect in a subgroup analysis (203, 204).

However, in spite of first efforts (205), the optimal regimen of timing, target organ, dosage and fractionation remains elusive and future trials and translational research need to address these important questions to maximize the potentially beneficial combination effects of radiotherapy and immunotherapy (206). The underlying molecular mechanisms are being investigated intensely and might lead to more promising designs for future clinical trials. PD-1 signaling has been linked to abscopal responses by knock-out and inhibition in *in vivo* models of stereotactic radiotherapy (207). The identification of radiation fractionation schedules leading to abscopal effects in combination with CTLA-4 blockade in an *in vivo* model of breast cancer was linked to the induction of cytosolic double-stranded DNA. With high radiation doses, the induction of the exonuclease TREX-1 degrading the DNA fragments, no abscopal effects were observed (208).

## Rationale for Selecting Patients With Hypoxic Tumors for Combination Treatment

**Figure 3:**
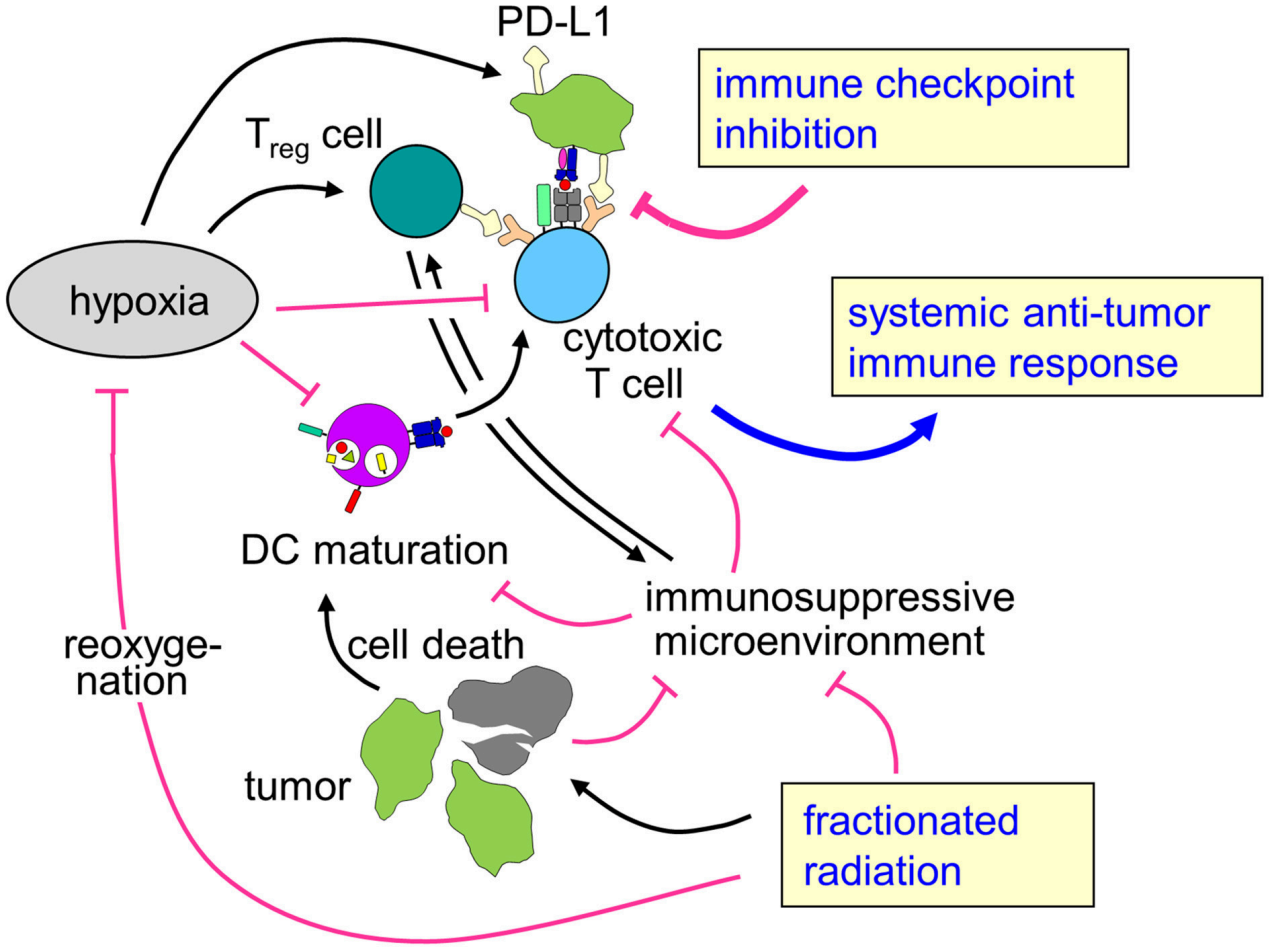
Rationale for combining radiotherapy and immune checkpoint inhibition to overcome therapy resistance of hypoxic tumors. Tumor hypoxia is a key player for the prognosis of cancer patients and resistance to radiotherapy and possibly also for anti-tumor immune response. Fractionated radiotherapy may lead to reoxygenation. The profound immune suppressive microenvironment (see chapter Immunosuppression in the hypoxic tumor microenvironment) predominantly in hypoxic tumors as well as upregulation of immune checkpoint molecules might hint at a rationale to combine fractionated radiotherapy with immune checkpoint inhibition in patients with hypoxic tumors to enhance local control and systemic anti-tumor immune effects.

To the best of our knowledge, there are no data on combined radiotherapy and immune checkpoint inhibition focusing on hypoxic tumors. However, as hypoxic tumors are intrinsically more radioresistant than normoxic counterparts and show reduced local control and higher rates of distant metastases, there is a specific clinical need in this subgroup of patients for more effective therapies. As hypoxia also leads to dramatically impaired anti-tumor immune responses, enhancing immune-mediated tumor control mechanisms might be a promising strategy, especially because the combination of immune checkpoint inhibition and radiotherapy has been described to improve local control as well as to induce abscopal effects leading to better systemic tumor control. The here described effects of hypoxia with increased mutational load and upregulation of immune checkpoints such as PD-L1 might even hint at improved responsiveness of hypoxic tumors to immune checkpoint inhibition, further strengthening the hypothesis that patients with hypoxic tumors might be a subgroup of specific interest for combination concepts of radiotherapy with immune checkpoint inhibition (Figure 3).

## Author Contributions

FE and SH designed the concept and wrote the manuscript. KZ wrote the chapter Rationale for combining radiotherapy and immunotherapy. SB wrote the chapter Treatment modifications targeting hypoxia in radiation oncology. DT, DZ, and all authors read and approved the manuscript.

### Conflict of Interest Statement

FE has a research collaboration with Merck KgAa. DZ, DT, KZ, SB, and FE have research and educational grants from Elekta, Philips, Siemens, Sennewald. SH has a research collaboration with Novocure.
